# Lung cancer screening: 360 degree review

**DOI:** 10.1186/1470-7330-15-S1-O13

**Published:** 2015-10-02

**Authors:** Christian J Herold, Theresa C McLoud

**Affiliations:** 1 Department of Biomedical Imaging and Image Guided Therapy, Medical University of Vienna / AKH, Vienna, Austria; 2 Department of Radiology, Massachusetts General Hospital / Harvard Medical School, Boston, Massachusetts, 02114, USA

## 

The most compelling evidence supporting the use of low dose computed tomography in the screening of high risk populations for lung cancer was generated by the National Lung Cancer Screening Trial carried out in the United States by the National Cancer Institute and the American College of Radiology Imaging Network.[1] This trial was a randomized prospective study which included over 53,000 participants. The two arms consisted of patients who were randomized to low dose CT and those who received standard radiography. Data from the NLST demonstrated that screening reduced mortality by 20% in the CT arm. Other smaller studies carried out in Europe have reported no mortality benefit. However, these studies included a younger screening population and had a smaller number of participants and probably did not have the power to show a mortality benefit.[2] Major medical societies and US government agencies such as US Preventative Services Task Force and now the Center for Medicare Services have now recommended LDCT. These decisions not only recommend screening but require US insurance companies and Medicare in the US to provide reimbursement.[3]

Some of the caveats of screening result from the limits of generalization of the NLST results based on risk groups and demographics. There are also potential harms and complications from the LDCT screening which include radiation exposure, over diagnosis, the use of invasive procedures for diagnosis and the high false positive rate. [1,4]

Lung cancer screening could have a strong impact on health care in Europe where there are over 268,000 lung cancer deaths per annum. Screening could save thousands of lives. However, lung cancer screening is currently not reimbursed and there are no screening programs accessible through health care systems or health plans. Important factors for lung cancer screening in Europe include refinement of inclusion criteria and stratification of risks, modeling of cost effectiveness data, and inclusion of evidence from European trials. Implementation of screening programs in Europe will require a collaborative effort among professional specialties, societies, organizations, and additional trial data.

The American College of Radiology has developed a reporting system for lung cancer screening designated “Lung Rads.” [5] It is a reporting system which is very similar to Bi-Rads that includes numeric categories. It associates CT findings with guideline based management decisions. LDCT findings are categorized from 1-4 according to the likelihood of malignancy. 1 and 2 are considered benign findings and 3 and 4 have a higher probability of malignancy. There are descriptions for each of the 4 categories plus an enumeration of findings. The categories include characterization of nodules including size and density (solid, part-solid, or non- solid ground glass). In addition to the categories and findings there are guidelines for management in each category with estimates of the probability of malignancy and the estimated population prevalence.

Constructing a lung cancer screening program in your institution presents many challenges. (6) Stake holders include the hospital leadership, referral providers, a multidisciplinary team, and support staff. Patient and physician outreach and education are essential. Standard protocols should be developed for image acquisition. Ideally image review should be performed by radiologists experienced in chest CT and nodule detection. Imaging software that combines CAD and volumetric measurements is desirable. Findings should be recorded according to the lung imaging reporting and data system such as Lung Rads. Communication of results to both the patient and physician is important and such communication should be stratified according to urgency.
